# Au nanodyes as enhanced contrast agents in wide field near infrared fluorescence lifetime imaging

**DOI:** 10.1186/s11671-024-03958-1

**Published:** 2024-01-25

**Authors:** Neelima Chacko, Menachem Motiei, Jadhav Suchita Suryakant, Michael Firer, Rinat Ankri

**Affiliations:** 1 Department of Physics, Faculty of Natural Science, Ariel University, 40700 Ariel, Israel; 2 Faculty of Engineering, The Institute of Nanotechnology and Advanced Materials, Bar-Ilan University, 5290002 Ramat Gan, Israel; 3 Department of Chemical Engineering, Faculty of Engineering, Ariel University, 40700 Ariel, Israel

**Keywords:** Fluorescence lifetime imaging, Gold nanospheres, Gold nanorods, Time-gated acquisition, Near infrared regime

## Abstract

The near-infrared (NIR) range of the electromagnetic (EM) spectrum offers a nearly transparent window for imaging tissue. Despite the significant potential of NIR fluorescence-based imaging, its establishment in basic research and clinical applications remains limited due to the scarcity of fluorescent molecules with absorption and emission properties in the NIR region, especially those suitable for biological applications. In this study, we present a novel approach by combining the widely used IRdye 800NHS fluorophore with gold nanospheres (GNSs) and gold nanorods (GNRs) to create Au nanodyes, with improved quantum yield (QY) and distinct lifetimes. These nanodyes exhibit varying photophysical properties due to the differences in the separation distance between the dye and the gold nanoparticles (GNP). Leveraging a rapid and highly sensitive wide-field fluorescence lifetime imaging (FLI) macroscopic set up, along with phasor based analysis, we introduce multiplexing capabilities for the Au nanodyes. Our approach showcases the ability to differentiate between NIR dyes with very similar, short lifetimes within a single image, using the combination of Au nanodyes and wide-field FLI. Furthermore, we demonstrate the uptake of Au nanodyes by mineral-oil induced plasmacytomas (MOPC315.bm) cells, indicating their potential for in vitro and in vivo applications.

**Graphical abstract:**

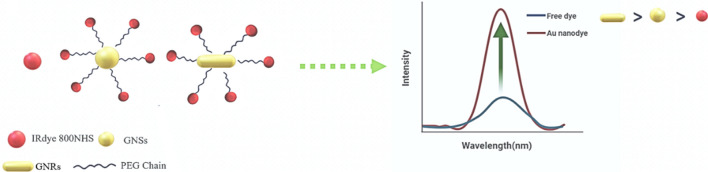

**Supplementary Information:**

The online version contains supplementary material available at 10.1186/s11671-024-03958-1.

## Introduction

Fluorescence lifetime imaging (FLI) offers several advantages, notably its reliance on the intrinsic fluorescence lifetime (FLT) of a fluorophore. The FLT, influenced by its molecular surroundings, provides insight into the biochemical state of intracellular or extracellular microenvironments. This characteristic has proven beneficial in diverse biomedical investigations, including microenvironment studies [1], molecular interaction monitoring [2], and improved multiplexing capabilities [3]. While FLI finds extensive use in in vitro research, its transition to in vivo imaging presents challenges due to limited visible light penetration through tissue and elevated background autofluorescence. These obstacles can be effectively addressed by leveraging near-infrared (NIR) or longer wavelengths in in vivo models. NIR fluorescence capitalizes on reduced photon scattering, light absorption, and autofluorescence, facilitating enhanced tissue penetration capabilities [4]. Despite the great advantages of NIR illumination, the NIR I range at 700–1000 nm is a relatively small spectral window that limits the possibilities for multiplexing because very few dyes absorb and emit in this range [5]. The lack of suitable NIR fluorophores possessing adequate quantum yield has significantly impeded advancements in this domain. Desirable properties of a fluorophore include high molar absorptivity and fluorescence quantum yield, resistance to photobleaching, good solubility in various solvents, and absorption and emission wavelengths suitable for the desired application. While the synthesis of novel NIR probes is a rapidly advancing realm, many preclinical investigations continue to rely on heptamethine dyes such as indocyanine green (ICG), FDA-approved with an 830 nm emission [6], and on the IRdye 800NHS, which emits at 810 nm and is at phase 2 of the clinical trials [7]. Despite their widespread use, these fluorophores suffer from low quantum yield (of 0.9% and 3.3% for ICG and IRdye 800NHS, respectively [8]. Additionally, their closely resembling fluorescence lifetimes [9], complicates their application in multiplexed FLI. Consequently, there is a real need for designing novel near-IR fluorophores, or refining existing ones, to attain elevated quantum yield and multiple fluorescence lifetimes, thus enhancing their utility.

In this work, we have synthesized biocompatible gold nanospheres and gold nanorods (GNSs and GNRs, respectively)-NIR dyes (named as *Au nanodyes*) with enhanced quantum yield and different FLTs, facilitating multiplexed FLI. Gold nanoparticles (GNPs) exhibit high absorption coefficients, augmenting their sensitivity in optical detection methods beyond conventional dyes [10]. Moreover, GNPs find widespread utility across various biomedical domains, including cancer therapy, drug delivery, and diverse biological applications, fostering their continued exploration [3, 11]. We conducted an investigation into the photophysical characteristics, including fluorescence emission, fluorescence lifetimes and quantum yield, of the biocompatible IRdye 800NHS and its conjugation with gold nanoparticles (GNPs). The proximity between metallic nanostructures and fluorophores plays a pivotal role in enhancing the fluorescence of the dye, as demonstrated in previous studies [12–14]. When the surface plasmon resonance (SPR) of the GNP aligns closely with the absorption peak of the dye, it results in heightened near-field effects, intensifying the absorption of light by the molecule [15, 16], termed as plasmonic enhancement. The most well known mechanisms involved in plasmonic enhancement are complex and can include the following phenomena: (i) Increased absorption and excitation; the localized surface plasmon resonance of the gold nanoparticle can enhance the absorption of light by the fluorophore, leading to increased excitation, (ii) Increased radiative decay; plasmonic nanoparticles can increase the radiative decay rate of the excited state, promoting fluorescence emission, and (iii) Decreased non-radiative decay; non-radiative decay pathways may be suppressed, favoring radiative decay and fluorescence emission. For these reasons, both the fluorescence intensity and lifetime of the fluorophore can be modulated by adjusting the distance between the dye and the Au nanostructures. This tuneable nature of the dye's fluorescence properties offers exciting possibilities for the creation of innovative NIR dyes tailored for fluorescence and fluorescence lifetime imaging in diverse biological and medical contexts [17]. This metal-fluorophore interaction hinges on parameters such as distance, excitation wavelength, metal size and shape, and dipole orientation [15, 18]. Electromagnetic coupling between the fluorophore and nanoparticle plasmon amplifies the radiative decay rate of the molecule at the emission wavelength [14], resulting with a change in the FLT of the dye, according to:

1 $$\tau = \frac{1}{{\Gamma_{{\text{R}}} + \Gamma_{{{\text{NR}}}} + k_{{\text{R}}} + k_{{{\text{NR}}}} }}$$

and the quantum yield (QY) increases by the additional radiative and non-radiative term arises due to the presence of metal ($\Gamma_{{\text{R}}} {\text{and}} \Gamma_{{{\text{NR}}}}$) at a specific distance from the fluorophore:

2 $${\text{QY}} = \frac{{\Gamma_{{\text{R}}} + K_{{\text{R}}} }}{{\Gamma_{{\text{R}}} + \Gamma_{{{\text{NR}}}} + k_{{\text{R}}} + k_{{{\text{NR}}}} }}$$

where ${\text{k}}_{{{\text{NR}}}}$ and ${\text{k}}_{{\text{R}}}$ and are the non-radiative and radiative decay rates, respectively, in the absence of the metal, and $\Gamma_{{\text{R}}} \;{\text{ and}}\; \Gamma_{{{\text{NR}}}}$ are the radiative and non-radiative decay rates, respectively, in the presence of metal [18].

In this study, the photophysical characterizations of IRdye 800NHS conjugated to GNSs and GNRs were studied. The GNRs exhibit SPR in the NIR, therefore are expected to significantly enhance the emission intensity of the IRdye 800NHS, through the plasmonic enhancement. The GNSs coupled with IRdye 800 were also explored, despite their SPR not falling within the NIR range, due to the widespread use of GNSs in clinical research, surpassing the usage of GNRs, and their comparatively simpler synthesis process. Investigating GNSs' impact on IRdye 800NHS is important, even with an expected low emission enhancement.

The GNSs and GNRs were coated with polyethylene glycol molecules (PEG, 1 kDa, 2 kDa and 7.5 kDa), yielding varying FLT and QY outcomes. The performance of these Au nanodyes in wide-field FLI was investigated using the state-of-the-art time-correlated SAPD array *SPAD512S* [19]. The uptake of Au nanodyes by mineral oil-induced plasmacytoma cells (MOPC315.bm) [19] was also investigated, paving the way for potential in vitro and in vivo applications.

## Materials and methods

### Gold nanospheres fabrication

The GNSs were synthesized as described in our previous publications [20, 21]. Briefly, the method of Enüstün and Turkevich was used to synthesize GNSs with a diameter of 20 nm [22]. 414 μL of 50% HAuCl_4_ was added to 200 mL distilled water, and then boiled. Upon boiling, 4.04 mL of 10% sodium citrate was added, and the solution was stirred for 5 min. The mixture was then kept at room temperature until was cooled down, then collected through repeated centrifugation processes. The gold nanoparticles were characterized using Transmission Electron Microscopy (TEM) and Ultraviolet–Visible (UV–VIS) spectroscopy. The concentration of synthesized gold nanospheres was determined to be 33.5 mg/mL through the utilization of Inductively Coupled Plasma Mass Spectrometry (ICP-MS) analysis. The gold nanoparticles (100 µL) were then conjugated to SH-PEG-NH_2_ molecules (creative PEG-Works, Winston Salem, USA) varying by their molecular weight (MW, 1,000,2000 and 7500 gr/mol), then they were covalently attached to the 5 µL of 1 mM stock solution IRdye 800NHS (Licor Bio, USA). The PEGylation of gold nanospheres (GNSs) were performed at room temperature through a continuous stirring of 2 h at room temperature, and the excess dye was removed through centrifugation. Calibration curves were generated by correlating fluorescence peaks with the concentrations of the free dye (IRdye 800NHS, see in Supplementary material, Fig. S1). Following incubation with GNPs and subsequent centrifugal separation, the concentration of residual free dye in each sample supernatant was gauged by measuring fluorescence emission. The calculation of dye loading on GNPs was accomplished by subtracting the initially added dye amount from the concentration of the remaining free dye [23], resulting with ~ 4 µM. Thus, the concentration of the dye before mixing nanoparticles was 5 µM, the concentration of the dye in supernatant was 1 µM, and final concentration of the dye on nanoparticle was 4 μM. The quantification of the GNSs concentrations post-centrifugation was conducted using ICP-MS, resulting with 3.3 mg/mL for the AuNSs samples.

### Gold nanorods fabrication

Gold nanorods were synthesised using a modified procedure of the seed-mediated growth method reported by Meneghetti et al. [24]. Gold seeds were prepared by stirring 250 µL of HAuCl_4_ (0.01 mM) with 9.75 mL of CTAB (0.1 mM). 600 μL of NaBH_4_ (0.01 M) were added to the seed solution, which is stirred for 10 min at room temperature. In a different flask, 95 mL of CTAB (0.1 M) was mixed with 5 mL of HAuCl_4_ (0.01 M). Then, 800 µL of silver nitrate (0.01 M) and 550 µL of ascorbic acid (0.1 M) were added to this mixture (CTAB and HAuCl_4_). 120 mL of the previously prepared seed solution was added to the flask, and the mixture rested overnight. The concentration of synthesized gold nanorods was determined to be 1.6 mg/mL through the utilization of Inductively Coupled Plasma Mass Spectrometry (ICP-MS) analysis The particles were concentrated by repetitive centrifugations until a clear suspension was achieved. Synthesised gold nanorods were PEGylated according to the procedure described above for the GNSs. The PEGylated GNRs were conjugated to 5 µL of 1 mM stock solution of IRdye 800NHS. The final concentration of the dye was calculated as described in the GNSs method section above. The quantification of GNRs concentrations post-centrifugation was conducted using ICP-MS, resulting with 0.1 mg/mL for the AuNRs samples.

### Steady-state fluorescence measurements

Absorbance, emission, and fluorescence lifetime (FLT) spectra were measured using the Fluorolog-Quanta Master (Horiba scientific, Japan). Data was analyzed using the FelixFL software (version 1.0.33.0, Horiba scientific, Japan). 1 cm optical path lengths quartz cuvettes were used for both absorbance and fluorescence measurements. For absorption and emission measurements, the samples were excited using a 75 W Xenon lamp, with a slit width of 10 nm and integration time of 1 s with a 0.1 nm step size in wavelength. The spectra of all synthesized AuNDs underwent normalization, wherein a ratio factor was computed by dividing the concentration of gold nanorods (1.6 mg/mL) by that of gold nanospheres (33.5 mg/mL), resulting in a ratio factor of 0.48. This calculated ratio factor was then multiplicatively applied to the intensities of the emission spectra associated with gold nanospheres conjugated to the IRdye 800NHS. The normalization process was designed to mitigate concentration-related variations, ensuring a coherent and comparable representation of the emission spectra for subsequent analyses and interpretations.

### Time correlated single photon counting (TCSPC) lifetime measurements

The lifetime measurements were performed using a delta diode of 830 ± 10 nm (a peak wavelength of 819 ± 10 nm), with a narrow 50 picoseconds (ps) pulse width, a 0.6 mW average power, and a 100 MHz repetition rate. The samples were placed in a 1 cm pathlength absorbance plastic cuvette. Instrument response function (IRF) was measured using LUDOX^®^ AS-30 colloidal silica (Sigma Aldrich, Israel) and an excitation wavelength of 805 nm through a 35 nm slit width. The fluorescence decay curves were analysed using the FelixFL decay analysis software (version 1.0.33.0, Horiba Scientific) based on a multiexponential model which involves an iterative reconvolution process. The quality of the fits was evaluated using the reduced χ^2^ value.

### Quantum yield calculations

The determination of the dyes' quantum yield involved the utilization of experimental data derived from absorption intensity and fluorescence lifetime measurements. The radiative lifetime (τ_r_) of the samples was computed using the Strickler–Berg equation [25]:

3 $$1/\tau_{r} = \frac{{\tilde{v}_{\max } }}{{3.42 \times 10^{8} }} \times n^{2} \smallint \varepsilon \left( {\tilde{\nu }} \right)d\tilde{\nu }$$

where *n* is the refractive index of the medium (in our measurements the medium was PBS), $\nu_{\max }$ is the absorption maximum (in wavenumbers, [cm^−1^]) and $\varepsilon$ is the absorption coefficient. $\varepsilon$ was determined using the Beer–Lambert law (Eq. 4), by substituting the values of the absorbance (*A*) from the absorbance spectra. *c* is the concentration of the dye and *l* is the pathlength of the cuvette (1 cm):

4 $$A = \varepsilon lc$$

The quantum yield of the samples was then determined by substituting the calculated values of τ_r_ and experimentally obtained fluorescence lifetimes (τ_r_ + k_nr_) into Eq. (3).

### Cells staining

MOPC315.bm cells were stained with IRdye 800NHS and Au nanodye according to the previously reported Carboxyfluorescein Diacetate Succinimidyl Ester (CFSE) labelling method [26]. 0.5 mg of the IRdye 800NHS (molecular weight: 1165.20 g/mol) were dissolved in 100 μL of DMSO. Cells were resuspended in 1 mL culture medium with a cells concentration of 5–10 million/mL. Thoroughly resuspend cells in the 1 mL of medium and place carefully in the bottom of a clean (non-wetted) 10 mL conical tube. Then add 110 µL of sterilized PBS to the non-wetted portion of the plastic at the top of the tube, ensuring it doesn’t make any contact with the cell solution. Resuspended 1 mL of the stock solution of IR dye in 110 µl of sterilized PBS. Quickly cap the tube and invert and vortex well to get a quick, uniform mixing of the solution. After that, incubate the cells for 15 min at room temperature and try to protect the tube from light. Wash the cells by diluting in 10 volumes of 5% (2 µL Of HI FCS and 40 µL of PBS) HI FCS following centrifugation at 20 °C at 300×*g* for 5 min. Then discard the supernatant. Count the number of cells using a hemocytometer (Trypan blue dye) and distribute the cells into Petri dishes with a cell concentration of 1milion in each for continuous analysis of a particular time period.

### Wide field FLI setup

The FLT imaging set up operated in the NIR regime, in the wavelengths range of 780–900 nm. A time-gated camera (SPAD512S, PI Imaging, Switzerland) has been used for wide field acquisition (overlapping 117 gate images, with a gate spacing of 397 ps). The excitation source was a fiber-coupled pulsed laser with a wavelength of 779 nm, 20 MHz repetition rate, and a pulse width of ~ 70 ps (VisIR-780, PicoQuant). The sample’s fluorescence was collected through a 808/25 long-pass (LP) emission filter (EM). The time-triggered in-pixel architecture enables time-resolved photon counting at a maximum rate of 97 kfps (1-bit frames). The photon detection efficiency of the detector was ~ 13% at 800 nm with a fill factor of 10.5% and a dark count rate with a median value of 7.5 Hz/pixel.

### Phasor based analyses

Phasor analyzes of the wide-field time-gated FLI data was performed as described in our previous paper [24], using a numerical code developed in our lab. In the basis of these programs, the phasor (*g*_*i,j*_*, s*_*i,j*_) of each pixel of coordinate (*i, j*) in the image is calculated according to:

5 $$\begin{array}{*{20}c} {g_{i,j} = \frac{{\mathop \sum \nolimits_{k = 1}^{N} F_{i,j} \left( {t_{k} } \right)\cos \left( {2\pi ft_{k} } \right)}}{{\mathop \sum \nolimits_{k = 1}^{N} F_{i,j} \left( {t_{k} } \right)}}} \\ {s_{i,j} = \frac{{\mathop \sum \nolimits_{k = 1}^{N} F_{i,j} \left( {t_{k} } \right)\sin \left( {2\pi ft_{k} } \right)}}{{\mathop \sum \nolimits_{k = 1}^{N} F_{i,j} \left( {t_{k} } \right)}} } \\ \end{array}$$

where *f* is the phasor harmonic (equal to the laser repetition rate = 1/*T*), k = 1…N is the gate number and $F_{i,j} \left( {t_{k} } \right)$ is the kth gate image value at pixel (i, j). When computing region of interest (ROI) phasor values, the $F_{i,j} \left( {t_{k} } \right)$ in Eq. (5)) are replaced by the sum of all $F_{i,j} \left( {t_{k} } \right)$ in the ROI. The FLT was calculated from:

6 $$\tau = \frac{1}{2\pi f}\frac{s}{g}$$

When the mean FLT was given by the mean of the phase lifetime histogram [27].

### Statistical analyses

All graphical representations were generated using GraphPad Prism version 9.2.0 and Origin Pro version 8. Data are presented as means ± standard deviation, derived from four repetitions of measurements conducted on the same sample.

## Results and discussion

### GNSs and GNRs characterization

**Fig. 1:**
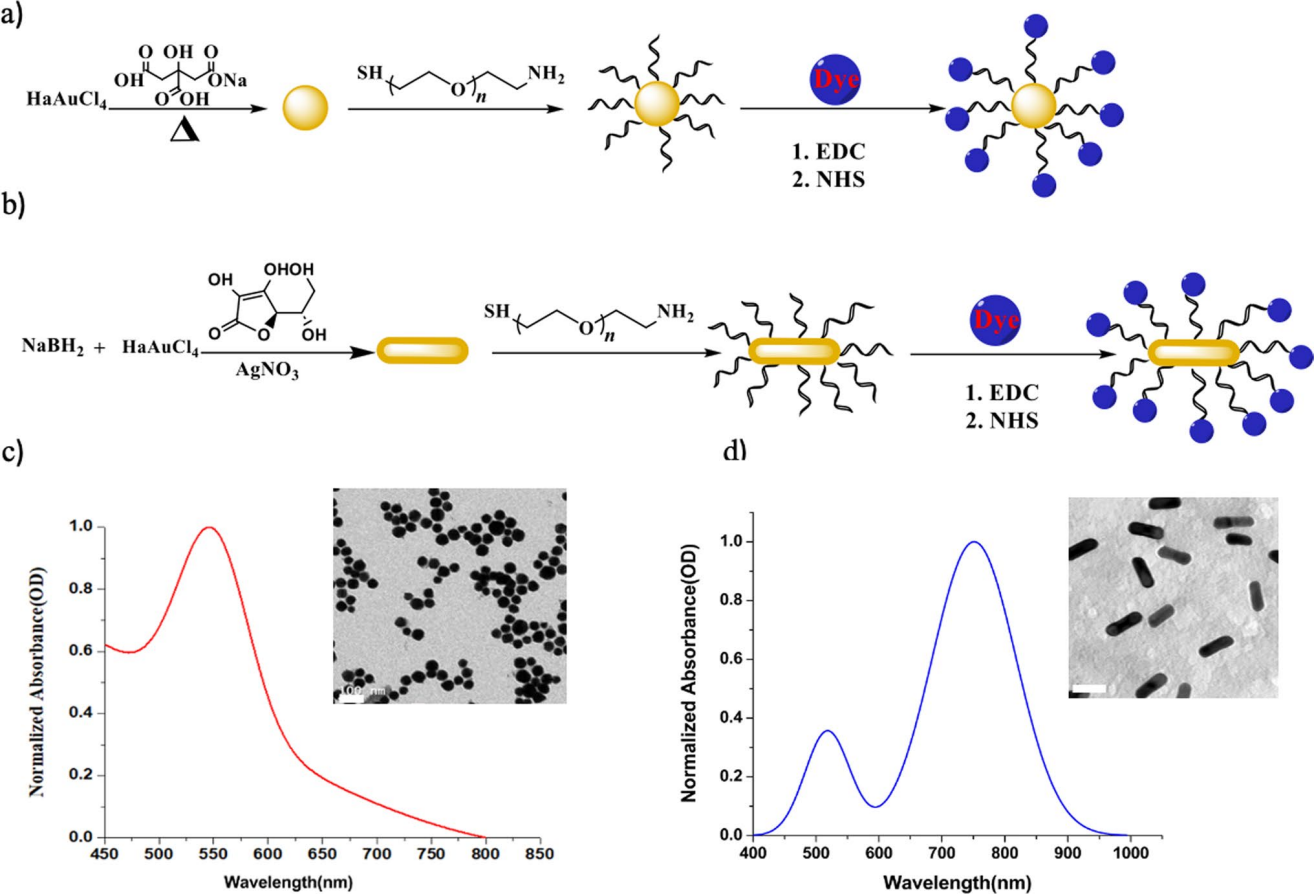
Synthesis and Characterizatio n of Au Nanoparticles. **a** Schematic representation of the GNSs synthesis process. **b** Schematic representation of the GNRs synthesis process. The conjugation of IRdye800 NHS fluorophores to pegylated GNPs was through an amide bond. **c** UV–Vis absorption spectrum of the GNSs. Inset: TEM image of the GNSs. Scale bar measures 100 nm. **d** UV–Vis absorption spectrum of the GNRs. Inset: TEM image of the GNRs. Scale bar measures 50 nm

Based on well-established protocols [20, 21, 24], GNSs and GNRs were synthesized and then conjugated with the IRdye 800NHS to form the Au Nanodyes, as shown in Fig. 1a, b. Figure 1c, d show the ultraviolet–visible (UV–Vis) spectrum of the GNSs and GNRs, with absorption maxima at 565 ± 0.05 nm and 770 ± 0.01, respectively. Three different PEG molecules were used for the IRdye 800NHS conjugation; 1 kDa, 2 kDa and 7.5 kDa, resulting with evaluated lengths of 6 nm, 12 nm and 45 nm [28], respectively. The size, shape and uniformity were characterized using a transmission electron microscopy (TEM), resulting with 40 nm and 25 × 65 nm in size for the GNSs and GNRs, respectively. The GNRs and GNSs emission spectra are also presented, in Figs. S2 and S3 in the Supplementary material file.

### Photophysical study of IRdye 800NHS and the Au nanodyes

**Fig. 2:**
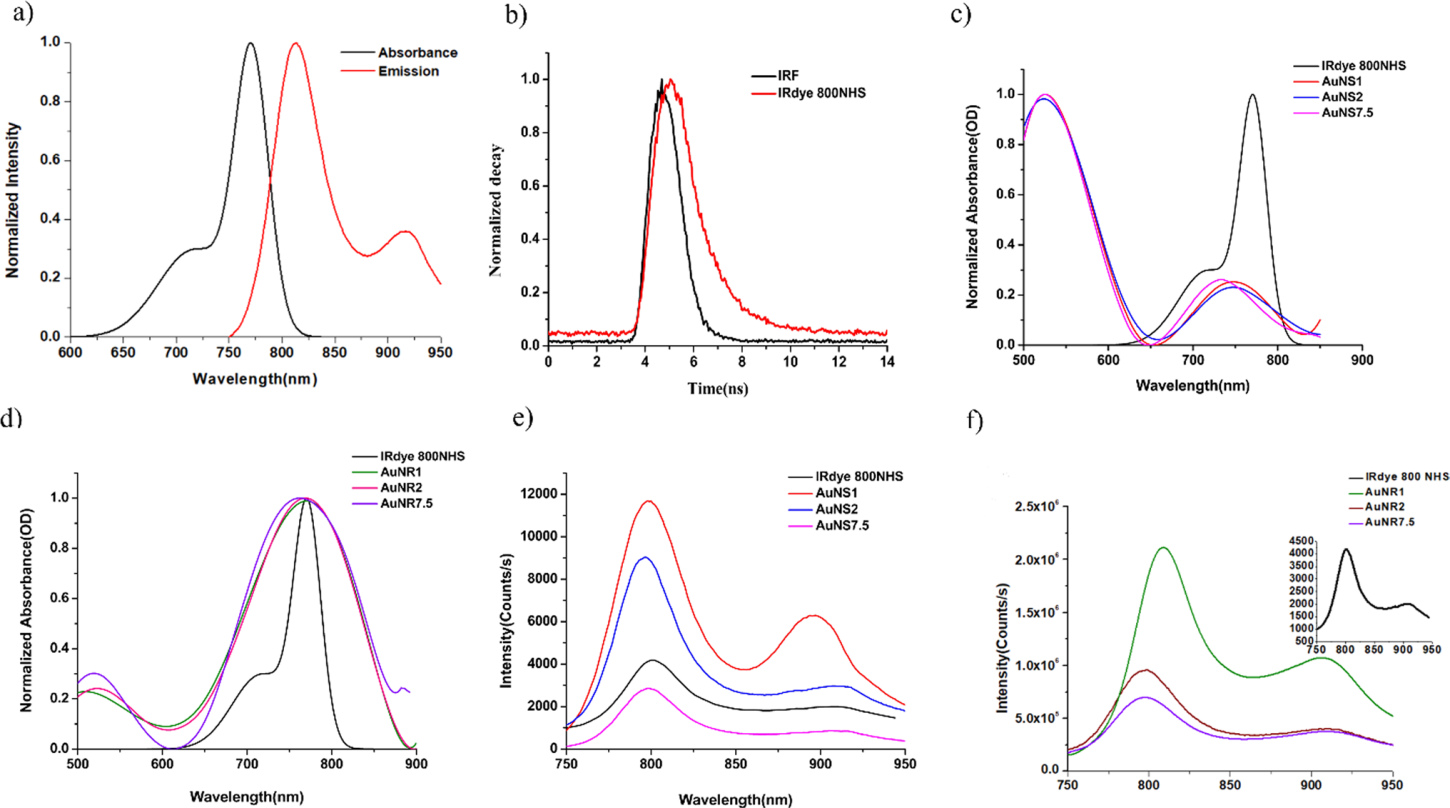
Photophysical characterization of IRdye 800NHS and Au nanodyes in PBS solution (pH 7). **a** Two-dimensional excitation-emission spectra of IRdye 800NHS. **b** Fluorescence decay rate of IRdye 800NHS. Black curve: IRF, red curve: IRdye 800NHS. **c** Absorption spectra of the Au nanospheres: AuNS1, AuNS2, and AuNS7.5. **d** Absorption spectra of the Au nanorods: AuNR1, AuNR2, and AuNR7.5. **e** Emission spectra of Au nanospheres: AuNS1, AuNS2, and AuNS7.5. PEG-IRdye 800NHS used as a control sample (black line). **f** Emission spectra of Au nanorods: AuNR1, AuNR2, and AuNR7.5. PEG-IRdye 800NHS (5 µM) used as a control sample (black line), and its emission can be seen in the inset. All samples were excited at 772 nm wavelength. AuNR1 exhibits the highest emission intensity among the Au nanodyes

Figure 2a illustrates the excitation-emission spectrum of IRdye800 NHS, indicating an excitation peak at 772 nm and an emission peak at 799 nm. The FLT of IRdye 800NHS was also measured, resulting with 0.67 ± 0.01 ns (Fig. 2b). The absorption and emission spectra of PEGylated GNSs and GNRs with varying molecular weights (1 kDa, 2 kDa, and 7.5 kDa) are presented in Fig. 2c, d after their conjugation to IRdye800 NHS, denoted as AuNS1, AuNS2, AuNS7.5, for the GNSs-, IRdye 800NHS and AuNR1, AuNR2, and AuNR7.5, for the GNRs- IRdye 800NHS compounds. The absorption peaks of AuNS1, AuNS2, and AuNS7.5 exhibited similarity to the GNSs absorption spectrum, with an additional peak around ~ 750 nm, indicating the binding of GNSs to IRdye800 NHS (Fig. 2c). AuNR1, AuNR2, and AuNR7.5 show a pronounced absorption peak at 763 nm, corresponding to the absorption peak of IRdye800 NHS. Figure 2d displays the emission spectra of Au nanodyes and free IRdye 800NHS dye in PBS solution, following excitation at 772 nm. Altered fluorescence intensities of Au nanodyes compared to free dye arise from modifications in the radiative decay rate due to the presence of metallic nanostructures, as described in Eqs. (1) and (2) above. Notably, AuNRs present significantly higher emission intensities, compare to the AuNSs. Enhanced fluorophore emission by metallic nanoparticles hinges on factors including size, shape, dielectric medium, and GNP-fluorophore distance [29–31]. The overlap between the localized surface plasmon resonance (LSPR) of the metal nanoparticle and the fluorophore’s spectral characteristics significantly influences fluorescence enhancement [23]. Thus, the proximity of IRdye800 NHS's excitation peak (779 nm) to gold nanorods’ maximum absorption (770 nm) compared to gold nanospheres (565 nm), results in higher fluorescence enhancement for AuNR1 compared to AuNS1 (Fig. 2d). Moreover, GNRs with shorter GNP-fluorophore distances (AuNR1) exhibit greater fluorescence enhancement potential compared to those with longer distances (AuNR2 and AuNR7.5), indicating that the fluorescence enhancement of Au nanodyes is distinctly influenced by GNP shape and its proximity to the fluorophore. Table 1 presents the emission intensity values for all samples.

**Table 1:** Fluorescence intensities of AuNDs measured from their fluorescence emission spectra

Dye	Fluorescence intensity (Counts/sec)
IRdye 800NHS	4.2 × 10^3^
AuNS1	1.2 × 10^4^
AuNS2	8.8 × 10^3^
AuNS7.5	2.8 × 10^3^
AuNR1	2.14 × 10^6^
AuNR2	9.5 × 10^5^
AuNR7.5	6.9 × 10^5^

### Au nanodyes fluorescence lifetime and quantum yield

**Fig. 3:**
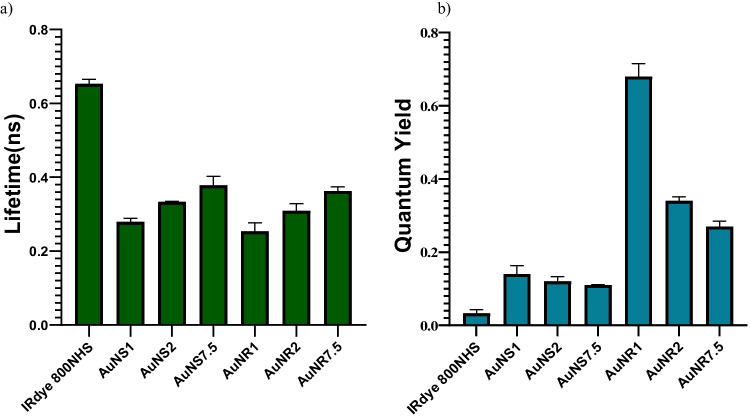
Fluorescence Lifetime and Quantum Yield Analysis of IRdye800 NHS and Au Nanodyes in PBS Solution. **a** TCSPC-based measurement of fluorescence lifetimes for both free dye and Au nanodyes. **b** Calculation of quantum yields (QY) using the Strickler–Berg equation, incorporating fluorescence lifetime and emission spectra. The QY calculation incorporated molar extinction coefficients for the Au nanodyes which is given in Table 2

Experimental results for the Au nanodye’s FLT was conducted using our TCSPC setup (as detailed in the *Methods* section above). The FLT values for both the Au nanodyes and IRdye 800NHS are depicted in Fig. 3. Initially, the inherent FLT of the free fluorophore was recorded, resulted with 0.67 ± 0.01 ns. Subsequent conjugation with GNSs and GNRs resulted in substantial FLT reductions, yielding 0.27 ± 0.01 ns, 0.33 ± 0.01 ns, and 0.37 ± 0.02 ns for AuNS1, AuNS2, and AuNS7.5, respectively, and 0.25 ± 0.02 ns, 0.30 ± 0.01 ns, and 0.36 ± 0.01 ns for AuNR1, AuNR2, and AuNR7.5, respectively (Fig. 3a). Figure 3b outlines the QY outcomes for both IRdye 800NHS and the Au nanodyes. QY values were computed by substituting the derived fluorescence emission intensity (radiative decay rate) and fluorescence lifetimes (radiative and non-radiative decay rates, as described above), into Eq. (2). The QY of the unbound IRdye 800NHS was determined as 0.03 ± 0.01, and it significantely increased in the presence of GNRs and GNSs. Specifically, the QY values for AuNS1, AuNS2, AuNS7.5, AuNR1, AuNR2, and AuNR7.5 were determined as 0.14 ± 0.02, 0.12 ± 0.013, 0.11 ± 0.001, 0.68 ± 0.035, 0.34 ± 0.011, and 0.27 ± 0.015, respectively. These variations in dye QY upon GNP conjugation can be attributed to the localized surface plasmon resonance (LSPR) of the metal nanoparticles, inducing local electric field enhancement which lead to elevated fluorophore excitation rates [21, 32].

**Table 2:** The molar extinction coefficients for the Au nanodyes were determined from the absorption spectra of the Au nanodyes

Dye	Molar excitation coefficient (1/M*cm)
IRdye 800NHS	240,000
AuNS1	160,000
AuNS2	162,800
AuNS7.5	161,000
AuNR1	524,000
AuNR2	768,600
AuNR7.5	335,800

### Wide field FLI with SPAD512S

**Fig. 4:**
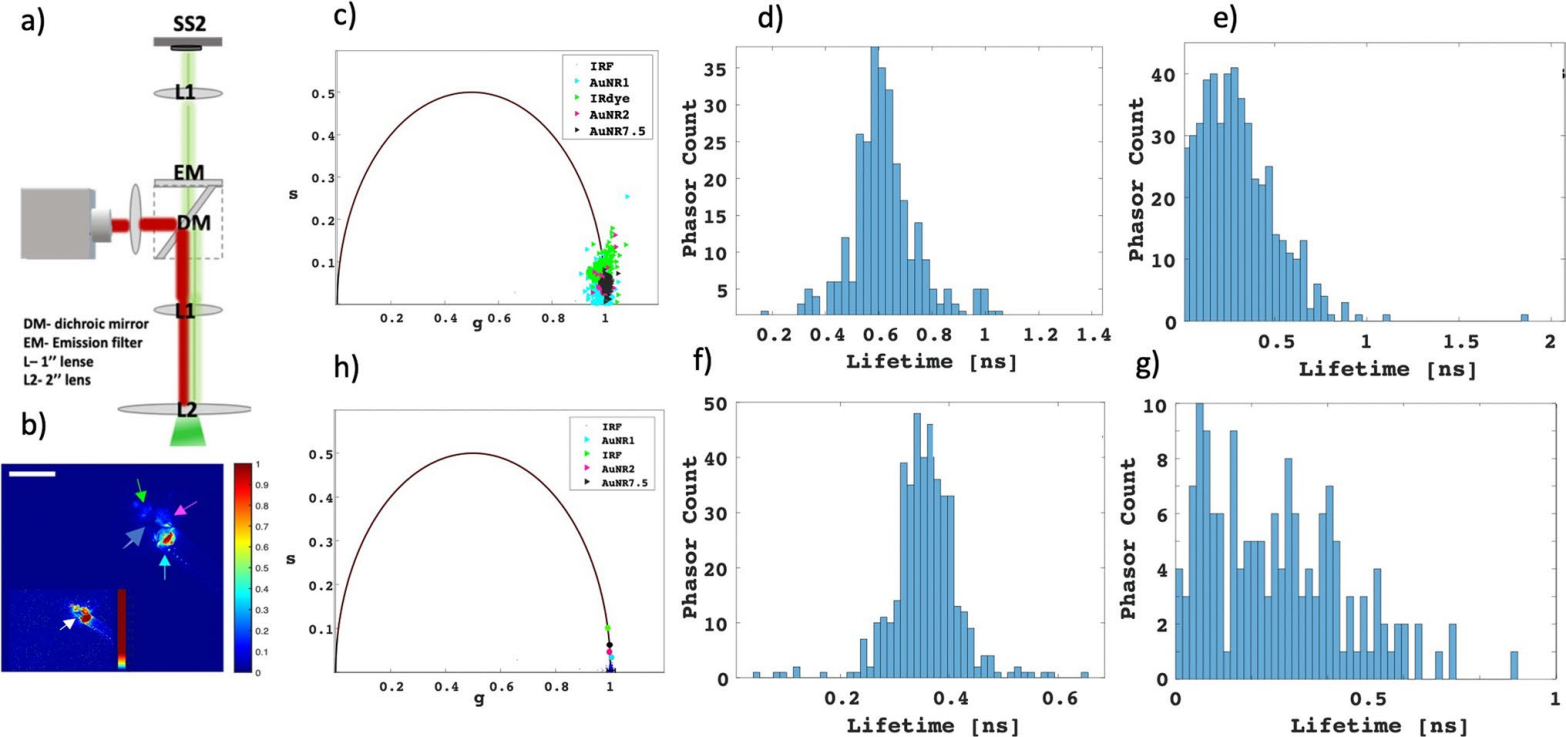
Multiplexed FLI of IRDye800 NHS, AuNR1, AuNR2 and AuNR7.5. **a** Experimental setup used for time-gated FLI: A 779 nm fiber-coupled picoseconds laser (Picoquant, Germany) illuminated the sample using wide-field mode through a 2″ NIR coated lens (L2, Newport, USA). Fluorescence emitted from the sample stage (SS) was collected via a 1″ NIR coated lens and a dichroic mirror (DM, Lahat technologies, Israel), and directed into the SPAD array through an emission filter (808 long pass filter, IDEX Health & Science, USA). Excitation power at focal plane: 5 mW, total integration time: 25 s. **b** Intensity image displaying the fluorescence from the different samples. Scale bar: 7 mm. Inset: same intensity image but featuring a different scale bar, to facilitate the visualization of AuNR7.5 fluorescence. **c** Phasor scatter plots for the four samples shown in (**b**). The colours of the different phasor spots are in accordance with the colours of the rows in panel b. Phasors were calibrated using the IRF lifetime (τ = 0 ns) and a phasor harmonic frequency f = 20 MHz. **d**–**f** Phase lifetime histograms corresponding to the phasor plots in (**c**). The histogram was Gaussian-fitted to extract a peak phase lifetime. The ROIs varied for the four samples, thus the phasor count values differ in the graphs. Background noise has been eliminated using consistent dark (no illumination) gated images. **h** The mean FLT values as was extracted from the scatter plots in panel c

Having characterized the lifetime behaviour of the IRDye800 NHS and the AuNDs, the samples were imaged using a highly sensitive time-gated SPAD camera [19]. The main objective of these experiments was to explore multiplexing FLI, using a single dye when conjugated to GNPs at different shapes and varying dye-GNP distances. A schematic illustration of the wide-field FLI setup utilized in our investigations is depicted in Fig. 4a. The fluorescence image of four representative dyes, IRDye 800NHS, AuNR1, AuNR2 and AuNR7.5, as captured by the SPAD512S, is shown in Fig. 4b. Phasor analyses of intensity spots in Fig. 4b are displayed in Fig. 4c, allow for the differentiation of the samples within a single image frame, based on their fluorescence lifetimes. Histograms detailing the number of phasor counts for each fluorescence lifetime were constructed from these phasor plots (Figs. 4d–g). The phase lifetimes were computed for each pixel within a region of interest (ROI) in each intensity spot (ROIs were not the same for each spot), furnishing data essential for constructing histograms that represent the count distribution for each phase lifetime. The mean of the histogram was used to calculate phase lifetimes for each individual dye. The resulting fluorescence lifetimes for different samples closely align with the TCSPC results illustrated in Fig. 3a, suggesting the following fluorescence lifetimes: 0.63 ns for IRDye 800NHS and 0.21 ns, 0.29 ns, 0.35 ns for AuNR1, AuNR7.5 and AuNR7.5, respectively. Figure 4e depicts the mean FLTs derived from these histograms, presented on the universal cycle. This visual representation serves as an effective means to distinguish between the various samples, despite the high proximity of the different FLT values. Similar results presenting the phasor analyses of IRDye 800NHS and AuNS are presented in Fig. S4 in the Supplementary material file.

These findings indicate that it is feasible to distinguish between the free dye and the AuNDs by extracting their FLTs through phasor-based analysis. In fact, the phasor spots on the universal circle (UC) are closely positioned, making visual differentiation challenging. However, by employing phasor histograms, distinct FLTs can be extracted. This enables the utilization of the same dye, when linked to GNPs, to exhibit varying FLTs in FLI.

### MOPC315.bm cells labeling and tracking

**Fig. 5:**
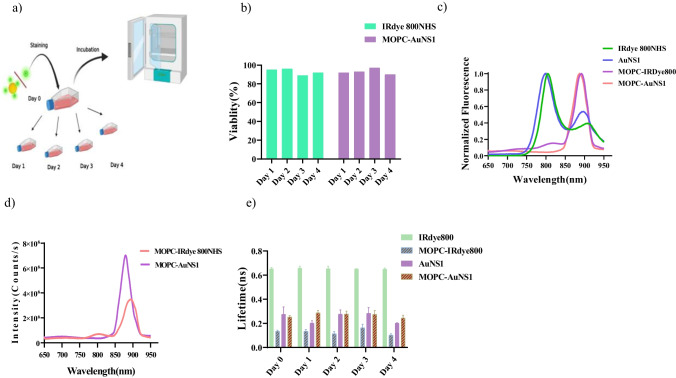
In Vitro Assessment of IRdye 800NHS and Au Nanodyes in MOPC315.bm Cells. **a** Illustration outlining the staining procedure and four-day tracking of MOPC315.bm cells using CFSE labelling with IRdye 800NHS and AuNS1. The stained cells were subjected to incubation and monitored over a span of four days. **b** Viability test results for MOPC315.bm cells stained with IRdye 800NHS and AuNS1, demonstrating sustained cell viability throughout the tracking period. **c** Normalized fluorescence emission spectra of IRDye800, AuNS1, and MOPC315.bm cells stained with both dyes. **d** Unnormalized fluorescence emission spectra of MOPC315.bm cells stained with both dyes. **e** Fluorescence Lifetime (FLT) measurements for the dyes and MOPC315.bm cells stained with the dyes

After thoroughly characterizing the photophysical properties of the various dyes, we proceeded to investigate their biocompatibility aspects. For this purpose, MOPC315.bm cells, a human multiple myeloma (MM) model [33], were employed. The MOPC315.bm cells were subjected to staining with AuNS1 and IRdye 800NHS, followed by an examination of the impact of these dyes on cell viability in vitro over a span of four days, as depicted in Fig. 5a. The results of viability assessments post the uptake of these dyes exhibited robust proliferation levels, indicating the biocompatibility of the dyes (Fig. 5b). Additionally, Fig. 5c illustrates the fluorescence emission spectra of the dyes employed in this study (specifically, IRdye 800NHS and AuNS1), alongside the spectra of the stained cells. Following dye uptake by the cells, a pronounced red shift in the emission spectra of the dyes (~ 91 nm) was observed (with the fluorescence emission peak shifting from 799 ± 1 to 890 ± 3 nm). This redshift can be attributed to the affinity of the IRdye 800NHS dye to bind to cell surface proteins [34]. Notably, MOPC315.bm cells stained with AuNS1 exhibited heightened fluorescence intensity compared to those stained with IRdye 800NHS (Fig. 5d, 3.5e6 and 7.6e6, respectively), which corresponded to the enhanced intensity of AuNS1 previously observed. Over the course of the four-day tracking period, the FLT values of the various samples were also measured, resulting in average values of 0.65 ± 0.03 ns and 0.24 ± 0.04 ns for IRdye 800NHS and AuNS1, respectively. MOPC315.bm cells labelled with IRdye 800NHS and AuNS1 exhibited average FLT values of 0.14 ± 0.02 ns and 0.27 ± 0.017 ns, respectively (Fig. 5e). FLI images of the labelled MOPC315.bm cells are presented in the Supplementary materials, Additional file 1: Figure S5.

The development of NIR contrast agents with adjustable lifetimes holds considerable importance. The key novelty in this study lies in the conjugation of GNPs with IRDye 800NHS, combined with FLI using a time-gated SPAD array and phasor analysis. While the conjugation of GNPs to IRDye 800 has previously been explored, primarily for applications like photothermal therapy, the novel aspect of this approach is its application in fine-tuning FLI.

In our research, we employed PEG chains of different lengths (1 kDa, 2 kDa, and 7.5 kDa) to synthesize distinct AuNDs, leading to varying distances between the dye and the GNPs. The outcomes revealed that the shorter was the PEG chain, the higher was the fluorescence intensity of the AuND (Fig. 2e, f). The shape of the GNPs also exerted a distinct impact on the fluorescence intensity. Our investigation encompassed two varieties of AuNDs: AuNSs and AuNRs. Notably, the AuNRs exhibited greater fluorescence emission compared to the AuNSs (Fig. 2e, f). This observation was in line with expectations, given that the GNRs possessed a localized surface plasmon resonance at 750 nm, which is in closer proximity to the absorption peak of IRdye 800NHS (at 779 nm) as compared to the absorption peak of the GNSs (at 565 nm).

Both FLT and QY of IRdye 800NHS underwent modifications due to different molecular weight PEG chains and the shape of the metallic nanostructure (Fig. 3a–b). The attachment of 1 kDa, 2 kDa, and 7.5 kDa PEG chains to gold nanoparticles resulted in alterations to the fluorescence lifetime. The fluorescence lifetime of IRdye 800 NHS with PEGylated gold nanoparticles was shorter than that of IRdye 800 NHS without PEGylated gold nanoparticles. This outcome aligns with the expectations outlined in Eq. (2) and is in line with the principles of metal-enhanced fluorescence or radiation decay. The presence of metallic nanostructures also led to changes in the quantum yield, with the highest quantum yield achieved through conjugation with 1 kDa PEGylated GNRs (Fig. 3b). In the case of AuNRs, the most likely explanation for the enhancement in QY is that the plasmonic field generated by the GNP impacts both the radiative and nonradiative decay rates of the dye [35], giving rise to varied lifetimes and improved quantum yields of the Au nanodyes. In scenarios where the GNP, like AuNSs, lack a plasmon resonance akin to the attached dye, the mechanism responsible for the enhanced quantum yield may differ from the MEF mechanism. We propose that this enhancement could be attributed to the improved stability of the dye upon conjugation to the gold nanoparticles. Previous reports indicate that stabilizing dyes from the Cyanine family, such as IRdye 800, can lead to a significant increase in QY. For instance, in the case of Cy3B, isomerization is prevented by chemical rigidization of the polymethine chain and, as a consequence, its fluorescence quantum yield is about ten times larger than that of Cy3 in aqueous solution [36, 37]. A similar phenomenon has been reported by Bardhan et al*.* where they suggest that the enhanced QY from 7 to 11% (increase of 1.6 times) of the IRdye 800NHS might result from its attachment to a protein, providing steric stability to the dye [31]. Still, in the above reported works, when the fluorophore undergoes chemical-steric stabilization, both QY and FLT increased. In our experiments on AuNSs, the results indicate an elevated QY but a decrease in FLT. We can infer that the heightened QY results from steric stabilization of the fluorophore by the GNS, when this steric stabilization is more complex than chemical stabilization, predicting a specific behavior for FLT becomes challenging. Further investigation is warranted to comprehensively understand this observed behavior.

Gold nanorods, characterized by a strong plasmon resonance in the near-infrared region, are promising probes for various applications. The transition of nanoparticles from a spherical to a rod-like configuration induces the emergence of new SPRs and introduces anisotropy [38]. GNRs exhibit two distinctive SPRs along their transversal and longitudinal axes. The longitudinal plasmon resonance enables the absorption of wavelength radiation in the NIR region [38, 39], while the transversal absorption peaks exhibit minimal changes with variations in the size of GNRs. Conversely, the longitudinal axis absorption peaks exhibit substantial alterations with the aspect ratio (length/width) of GNRs [40]. This anisotropic nature introduces several challenges. Firstly, their orientation sensitivity to incident light, determined by the nanorod's longitudinal axis, can impact the consistency of measurements and imaging results. Secondly, achieving monodispersity in size and shape is difficult, leading to variations that affect the reproducibility of experiments. Thirdly, anisotropic nanoparticles may have limited targeting efficiency and face challenges in surface functionalization compared to spherical counterparts. These issues must be carefully addressed to optimize the use of gold nanorods in biomedical applications, especially in imaging and therapeutics.

Figure 4 illustrates the feasibility of FLI using a single dye, which is conjugated to GNPs at different GNP-dye distances. The phasor analyses of intensity-based images are showcased, demonstrating a straightforward and distinct differentiation of the four dyes within a single image. The phasor scatter plots were generated by calculating the phasor for each pixel within the region of interest (ROI) for all four samples shown in panel b. Although there is overlap among the spots, primarily arising from the proximity in the FLTs of the distinct samples, each spot corresponds to a distinct mean value. This is evident from the histograms presented in panels d-f. A graph displaying the resulting mean FLT values from the phasor scatter plots (panel h in Fig. 4) shows distinct positions of the FLTs on the universal cycle, even if they are in proximity due to similarities in their FLT values.

The subsequent step involved utilizing PEGylated AuNS1 for in vitro studies with MOPC315.bm cells in the NIR region. The three PEG chains of 1 kDa, 2 kDa, and 7.5 kDa employed resulted in a shorter fluorescence lifetime and higher quantum yield of the dye due to changes in its radiative decay rates (Fig. 3b). Notably, prior researches [23, 31, 35, 41] on fluorescence enhancement of IRdye 800NHS by gold nanoparticles did not delve into in vitro experiments. In our study, we investigated the behavior of IRdye 800NHS and Au nanodye in MOPC315.bm cells in vitro. It was found that both IRdye 800NHS and Au nanodye had no adverse effects on cell viability. This biocompatibility was demonstrated by tracking labeled cells over a four-day period. In the presence of MOPC315.bm cells, the stability of fluorescence lifetime for Au nanodye exhibited only insignificant alterations compared to IRdye 800NHS. Moreover, we observed a comparable fluorescence enhancement in cells stained with Au nanodye as compared to those stained with IRdye 800NHS. GNPs were functionalized with polyethylene glycol chains to improve biocompatibility and promote cellular uptake [42]. Extensive in vitro studies consistently show an optimal range for cellular uptake, typically in the 10–60 nm diameter range for GNPs [43]. Cell viability tests (Fig. 5b), indicating that the IRdye 800 NHS and Au nano dye were not toxic to the cells and did not have a negative effect on the MOPC315.bm cell’s viability. This discovery introduces a promising avenue for biomolecular imaging in the near-infrared range through the utilization of metal-enhanced fluorescence.

Our findings demonstrate the superior photophysical properties of AuNRs in comparison to AuNSs. Consequently, in future in vitro and in vivo experiments, the preference will be given to AuNRs, as was also proffered in the wide field imaging given in Fig. 4 (which uses AuNRs). The in vitro part in this manuscript focuses on presenting a proof of concept regarding the potential uptake of AuNPs by cancer cells without altering cell viability or the FLTs of the dyes. This proof of concept is anticipated to be independent of the shape of the GNPs, thus applicable to AuNRs as well. This kind of proof was also achieved in our paper from 2022, in which Ankri et. al research involved the utilization of gold nanospheres for in vitro studies, aimed at comprehending the impact of gold nanoparticles on cells [44]. Subsequently, in vivo studies were conducted using gold nanorods, suggesting that the structural variations in gold nanoparticles did not have a discernible effect on both the viability and attachment of the nanoparticles to cells.

## Conclusions

This study introduces Au nanodots as contrast agents emitting within the NIR I window. By utilizing distinct morphological gold nanoparticles (GNSs and GNRs) conjugated with the near-infrared dye IRdye 800NHS, we investigate the interaction between GNPs and the dye, achieving modulation of NIR fluorescence emission intensity and lifetime. Unlike previous approaches primarily employing long-lifetime lanthanides [45, 46] for NIR fluorescence/luminescence lifetime imaging, especially in wide field imaging, our work presents a novel class of NIR fluorescence lifetime contrast agents with both short fluorescence lifetimes and ease of synthesis. These unique characteristic holds promise for applications such as image-guided surgery, offering rapid feedback due to the picosecond-scale fluorescence lifetimes of the AuNDs.

To conclude, this paper presents a study on the interaction between AuNDs and the NIR dye; IRdye 800NHS. The synthesis of these contrast agents is straightforward and highly effective from a chemical point of view. AuNPs, IRdye 800CW, and PEGs are commercially available and clinically relevant, which may accelerate the future clinical translation. There might be a concern on the controllability of the distance between AuNP and NIR dye because PEG is a flexible chain. For this purpose, in future studies we will conjugate between the dye and the GNPs through DNA strands, which should present a more stable distance.

### Supplementary Information

Supplementary file1 (DOCX 1930 KB)

## Data Availability

The data in this work are available in the manuscript or available from the corresponding author upon reasonable request.
