# Supplementary material for: Au nanodyes as enhanced contrast agents in wide field near infrared fluorescence lifetime imaging
Source: Discov Nano. 2024 Jan 25;19(1):18. doi: 10.1186/s11671-024-03958-1 (PMC10810770; doi:10.1186/s11671-024-03958-1)
Supplement: Supplementary file 1 — Supplementary file1 (DOCX 1930 KB) [file 11671_2024_3958_MOESM1_ESM.docx]

**Supplementary material:** **Au Nanodyes as Enhanced Contrast Agents in Wide Field Near Infrared Fluorescence Lifetime Imaging**

Neelima Chacko^1^, Menachem Motiei^2^, Jadhav Suchita Suryakant^3^, Michael Firer^3^, and Rinat Ankri*^1^

1. Department of Physics, Faculty of Natural Science, Ariel University, Ariel 40700, Israel
2. Faculty of Engineering & The Institute of Nanotechnology and Advanced Materials, Bar-Ilan University, Ramat Gan 5290002, Israel
3. Department of Chemical Engineering, Faculty of Engineering, Ariel University, Ariel 40700, Israel

*Corresponding author: [rinatsel@ariel.ac.il](mailto:rinatsel@ariel.ac.il)

**Content:**

Figure S1: Calibration curve of fluorescence peak versus free dye concentration.

Figure S2: Emission Spectra of GNRs in PBS (pH 7) excited at 700nm

Figure S3: Emission Spectra of GNSs in PBS (pH 7) excited at 700nm

Figure S4: Multiplexed FLI of IRDye800 NHS, AuNS1, and AuNS7.5.

Figure S5: FLI of MOPC315.bm following IRDye 800NHS and AuNS1 uptake.


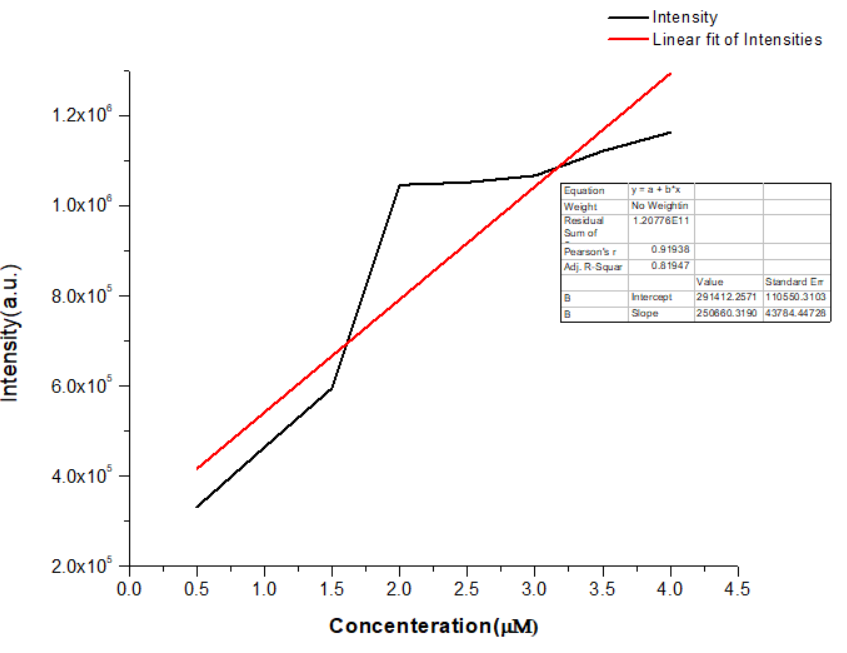
Figure S1: Calibration curve of fluorescence peak versus free dye concentration.

The concentration of remaining free dye in each sample supernatant, following incubation with the GNPs and centrifugal separation, was derived by measuring the fluorescence emission of the supernatants. The dye loading on GNPs was calculatedby subtracting from the amount of dye initially added. After conjugation, the fluorophore concentration was ~ 4 µM. (We took 5µL of 1mM stock solution of dye for conjugation with GNPs. After conjugation, the expected concentration is 5 µM.)

The calibration procedure was as follows: First, we constructed a calibration curve plotting concentration against intensity for the dye within the range of 0.5-4 µM. This range was chosen in consideration of the maximum dye concentration employed in the experiment, which was 5 µM (utilizing 5 µL of a 1 mM stock solution in 1 mL). Subsequent to the conjugation process, we measured the fluorescence intensity of the unconjugated dye and applied this value to the linear equation (y = mx + c) obtained from the calibration curve. In this equation, x represents concentration, y represents intensity, and m and c denote the slope and constant, respectively. By subtracting the initial dye concentration from the obtained concentration of unconjugated dye, we observed a minimal concentration of unbound dye. This outcome indicates that a significant proportion of the dye particles had successfully conjugated to the Au nanoparticles, underscoring that the bound concentration consistently remains lower than the free dye concentration. The consistently lower concentration of bound dye compared to free dye further emphasizes the efficacy of the conjugation process.


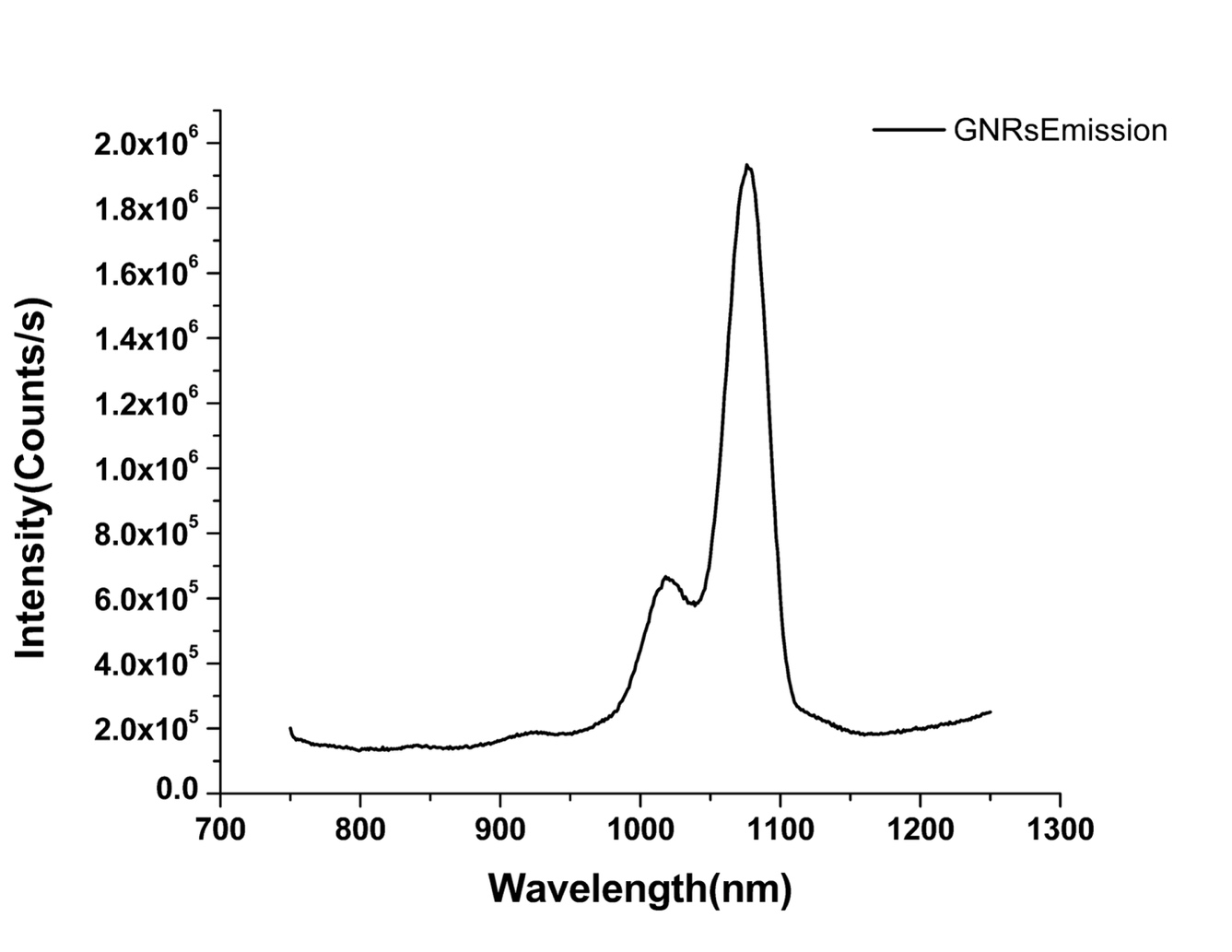
Figure S2: Emission Spectra of GNRs in PBS (pH 7) excited at 700nm


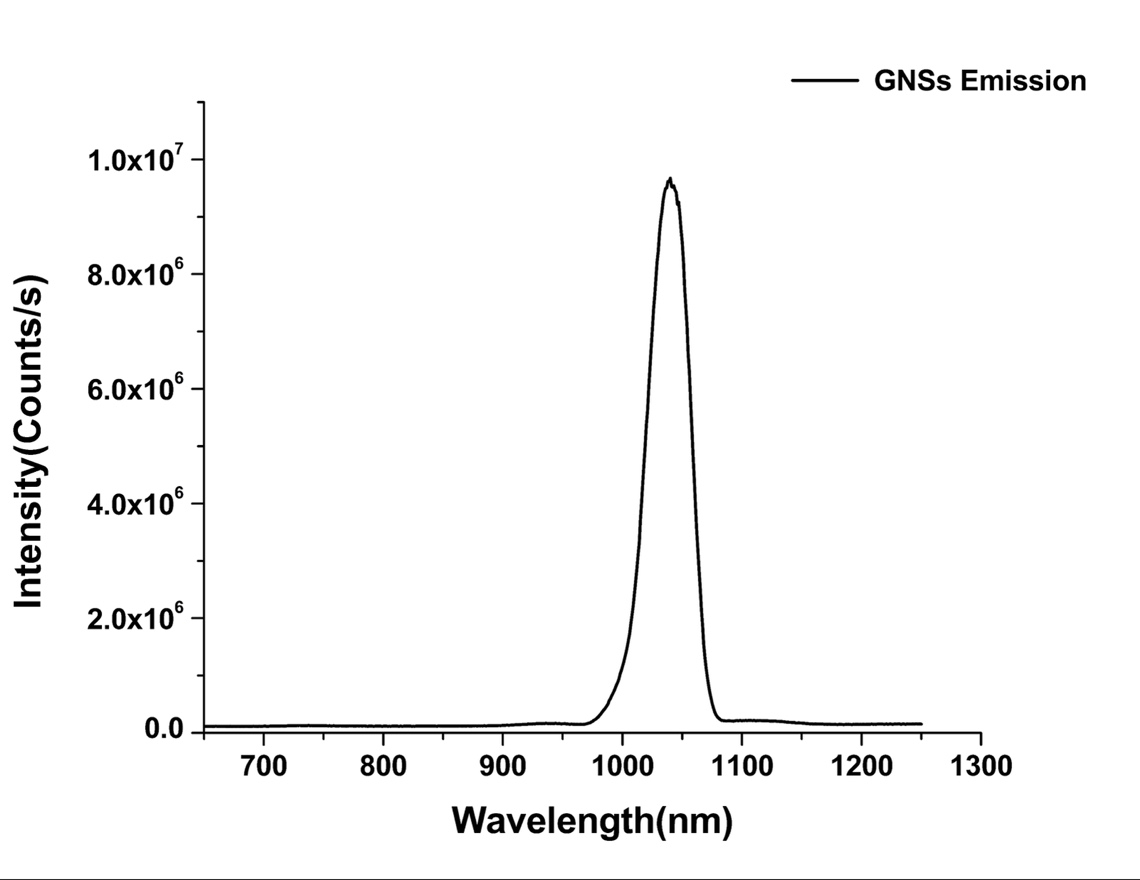
Figure S3: Emission Spectra of GNSs in PBS (pH 7) excited at 700nm
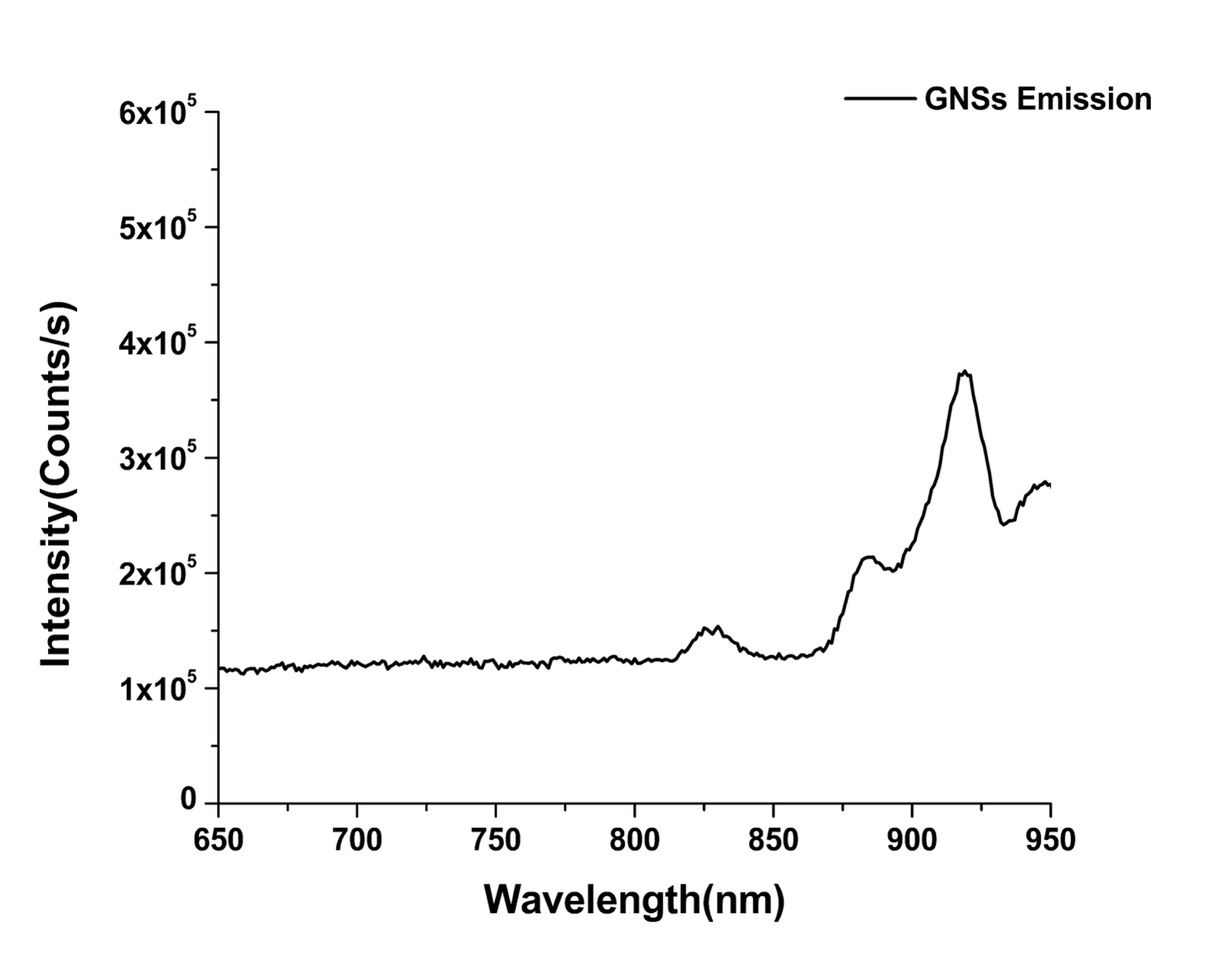


Figure S4: Multiplexed FLI of IRDye800 NHS, AuNS1, and AuNS7.5.

(a) Intensity image displaying AuNS7.5 (top right, blue circle), IRdye 800NHS (top left, pink circle), and AuNS1 (bottom, green circle). Scale bar: 7 mm. (b) Phasor scatter plots for the three dots shown in (a). Phasors were calibrated using the IRF lifetime (τ = 0 ns) and a phasor harmonic frequency f = 20 MHz. (c-e) Phase lifetime histograms corresponding to the phasor plots in (c). The histogram was Gaussian-fitted to extract a peak phase lifetime. The ROIs varied for the three samples, thus the phasor count values differ in the graphs. Background noise has been eliminated using consistent dark (no illumination) gated images.

Figure S5: FLI of MOPC315.bm following IRDye 800NHS and AuNS1 uptake.

\

**a)** The intensity image of the cells following the dyes uptake. **(b)** Phasor scatter plots for the three dots shown in (a). The phasors are calibrated using the IRF lifetime (τ = 0 ns) and a phasor harmonic frequency f = 20 MHz. **(c-d)** Phase lifetime histogram corresponding to the phasor plots shown in
